# Supplementary material for: Detection of antibodies to the SARS-CoV-2 spike glycoprotein in both serum and saliva enhances detection of infection
Source: medRxiv. 2020 Jun 18:2020.06.16.20133025. Preprint. [Version 1] doi: 10.1101/2020.06.16.20133025 (PMC7310662; doi:10.1101/2020.06.16.20133025)
Supplement: Supplement 2020 [file 86305-2020.06.16.20133025-1.docx]

Supplementary table I. Characteristics of samples tested

| ***Figure*** | ***Sample Type and Number*** | ***PCR status*** | ***Time from Symptoms Onset (Min-Max), days (d)*** |
| --- | --- | --- | --- |
| ***1*** | HS: N=6 | Positive | 14 (7-28) |
|  | NHC: N=6 | 5 postive; 1 no swab available | 23 (13-30) |
|  | AS: N=6 | Positive | N/A |
|  | Pre19: N=6 | N/A | N/A |
| ***2*** | HS: N=9 | Positive | 14 (5-32) |
|  | Pre19: N=9 | N/A | N/A |
| ***3*** | HS: N=3 | Positive | 21 (16-24) |
|  | NHC: N=3 | Positive | 17 (14-28) |
|  | Pre19: N=2 | N/A | N/A |
| ***4*** | NHC: N=30 | 29 positive; 1 no swab available | 14 (7-25) |
|  | Pre19: N=8 | N/A | N/A |
| ***5*** | SRSS: N=8 | PCR not performed | 24 (14-32) |
|  | Pre19: N=4 | N/A | N/A |
| ***6*** | UHB HCW serum and saliva N = 39 | Negative | N/A |

HS: Hospitalized subjects, NHC: Non-hospitalized Convalescent, AS: Asymptomatic Subject, SRSS: Self-reported Symptomatic Subject, UHB HCW: University Hospital Birmingham Healthcare Worker Study Subject; pre19: Pre-2019 subjects N/A: Not applied
